# Predictive Potential of Biomarkers of Intestinal Barrier Function for Therapeutic Management with Teduglutide in Patients with Short Bowel Syndrome

**DOI:** 10.3390/nu15194220

**Published:** 2023-09-29

**Authors:** Janine Büttner, Elisabeth Blüthner, Sophie Greif, Anja Kühl, Sefer Elezkurtaj, Jan Ulrich, Sebastian Maasberg, Christoph Jochum, Frank Tacke, Ulrich-Frank Pape

**Affiliations:** 1 Charité—Universitätsmedizin Berlin, Corporate Member of Freie Universität Berlin and Humboldt Universität zu Berlin, Department of Hepatology and Gastroenterology, Campus Charité Mitte and Campus Virchow Klinikum, 10117 Berlin, Germany; elisabeth.bluethner@charite.de (E.B.); sophie.greif@charite.de (S.G.); christoph.jochum@charite.de (C.J.); frank.tacke@charite.de (F.T.); 2 Berlin Institute of Health (BIH), 10178 Berlin, Germany; 3 iPATH.Berlin, Core Unit der Charité—Universitätsmedizin Berlin, Corporate Member of Freie Universität Berlin and Humboldt, Campus Benjamin Franklin, 12203 Berlin, Germany; anja.kuehl@charite.de; 4 Charité—Universitätsmedizin Berlin, Corporate Member of Freie Universität Berlin and Humboldt Universität zu Berlin, Department of Pathology, Campus Mitte, 10117 Berlin, Germany; sefer.elezkurtaj@charite.de; 5 Department of Internal Medicine and Gastroenterology, Asklepios Klinik St. Georg, 20099 Hamburg, Germany; ja.ulrich@asklepios.com (J.U.); s.maasberg@asklepios.com (S.M.)

**Keywords:** short bowel syndrome, chronic intestinal failure, glucagon-like peptide-2, teduglutide, intestinal permeability

## Abstract

Introduction: The human intestinal tract reacts to extensive resection with spontaneous intestinal adaptation. We analyzed whether gene expression analyses or intestinal permeability (IP) testing could provide biomarkers to describe regulation mechanisms in the intestinal barrier in short bowel syndrome (SBS) patients during adaptive response or treatment with the glucagon-like peptide-2 analog teduglutide. Methods: Relevant regions of the GLP-2 receptor gene were sequenced. Gene expression analyses and immunohistochemistry were performed from mucosal biopsies. IP was assessed using a carbohydrate oral ingestion test. Results: The study includes 59 SBS patients and 19 controls. Increases in gene expression with teduglutide were received for sucrase-isomaltase, sodium/glucose cotransporter 1, and calcium/calmodulin serine protein kinase. Mannitol recovery was decreased in SBS but elevated with teduglutide (Δ 40%), showed a positive correlation with remnant small bowel and an inverse correlation with parenteral support. Conclusions: Biomarkers predicting clinical and functional features in human SBS are very limited. Altered specific gene expression was shown for genes involved in nutrient transport but not for genes controlling tight junctions. However, mannitol recovery proved useful in describing the absorptive capacity of the gut during adaptation and treatment with teduglutide.

## 1. Introduction

Short bowel syndrome (SBS) is a rare disorder caused by extensive surgical resection or congenital diseases of the small intestine, resulting in decreased intestinal length and compromised function. Causes for resections could be vascular diseases, neoplasms, trauma, adhesion-related complications or inflammatory bowel disease. Patients with SBS comprise a heterogeneous population differing in small bowel length, anatomical portion, function of the remnant bowel, and presence of a colon in continuity. The severity of the disease is widely variable, from single micronutrient malabsorption to complete intestinal failure [1,2,3].

The human intestine reacts to resections with spontaneous adaptation. This adaptation involves expansion of the mucosal surface area and modulation of intestinal blood flow, secretions, permeability and motility that together increase absorptive capacity. The degree of intestinal adaptation after large resections depends on the extent of resection, the remaining intestinal anatomy, the luminal stimulation by enteral nutrients, and intestinotrophic factors [4,5,6].

Among the intestinotrophic growth factors, glucagon-like peptide-2 (GLP-2) has increasingly moved into the focus of current research [7]. Multiple actions for GLP-2 are described for the gastrointestinal mucosal epithelium, such as inhibited gastric acid secretion, enhanced carbohydrate transport, increased cell proliferation, decreased apoptosis, motility, and permeability [8,9]. GLP-2 expression is more prominent in distal sections of the small bowel and in the colon. The bioavailability of GLP-2 is controlled by renal clearance and enzymatic cleavage and has a short half-life of 7 min, after which it is degraded by the dipeptidyl peptidase IV. 

The actions of GLP-2 are transduced via a single G protein–coupled receptor (GLP-2R), whose expression is restricted to subepithelial myofibroblasts and subsets of enteric neurons and enteroendocrine cells [10]. Expression of GLP-2R mRNA was more detectable in jejunal and duodenal regions than in segments of the stomach, ileum or colon, suggesting a mainly endocrine mechanism of GLP-2 action [11,12]. 

The first clinically available drug in SBS was the GLP-2 analog teduglutide with an elongated half-life of up to two hours. Teduglutide has been shown to improve intestinal structural and functional integrity, followed by enhanced fluid and nutrient absorption in patients with SBS [13,14,15,16,17,18,19].

Currently, the mechanisms of intestinal barrier regulation and permeability (IP) during adaptive response or treatment with GLP-2 analogs are not well understood, and studies in human samples are scarce. Furthermore, biomarkers to predict either spontaneous intestinal adaptation, response to GLP-2 analog treatment, or to assist with the clinical management of GLP-2 treatment are very limited. 

At present, only two biomarkers for adaptation and treatment response are used in clinical practice. Firstly, parenteral support itself is used to classify chronic intestinal failure into functional categories based on the volume or energy content of parenteral nutrition [3,20]. It has also been shown that clinical response to treatment with GLP-2 analogs is characterized by a relevant reduction in parenteral requirements. The reduction of volume and/or days of parenteral support is one of the main goals of treatment, and patients with >20% reduction in parenteral support volume from baseline have been classified as teduglutide responders [21]. Secondly, the measurement of citrulline in plasma may be used as a marker of enterocyte mass. A first study by Crenn et al. revealed reduced citrulline concentrations in patients with SBS; the extent of citrullineamia correlated with the remaining small bowel length [22], but it is not routinely used [16]. 

GLP-2, as an intestinotrophic enteroendocrine peptide, is supposed to regulate the barrier function via the tight junctions of the enterocytes. Tight junctions are intercellular permeability seals that regulate paracellular transport across epithelia. The relationship between GLP-2 tight junction protein expression and tight junction function was studied by Moran et al. in a Caco-2 colonic cell culture model [23]. GLP-2 exposed to Caco-2 cell monolayers was shown to induce a relevant increase in transepithelial electrical resistance compared to untreated control cells. At the same time, expression of the tight junction proteins occludin and zona occludens-1 (ZO-1) was increased, while no changes were seen for claudin-1 and claudin-4. 

Based on this study, we aimed to investigate the effect of the GLP-2 analog teduglutide on tight junction gene expression and/or the regulation of intestinal permeability (IP) in humans.

Furthermore, we analyzed whether measurement of IP or whether gene expression analyses of genes likely associated with IP not necessarily limited to tight junction-associated genes can be useful biomarkers to describe regulatory mechanisms in the intestinal barrier in patients with SBS with or without teduglutide treatment.

## 2. Materials and Methods

### 2.1. Study Individuals

The study was approved by the ethics committee of the Charité—Universitätsmedizin Berlin (EA2/245/18), and informed consent was obtained from each participant. From 2019 to 2022, 59 SBS patients and 19 healthy controls were recruited from the intestinal failure clinic at Charité—Universitätsmedizin Berlin (Berlin, Germany). Diagnosis of SBS/chronic intestinal failure was based on standard clinical criteria [20]. SBS patients were included if they fulfilled the following criteria: extensive intestinal resection leading to chronic intestinal failure requiring intermittent or continuous partial or complete parenteral nutrition for calorie and/or volume administration. Malignant causes, as well as patients under the age of 18 and palliative patients due to other than malignant causes, were excluded from the study, as well as patients who had not consented. Healthy control values were obtained from adult, healthy volunteers. Clinical data of SBS patients were obtained through retrospective collection from the patients’ clinical charts. The following data of SBS patients were obtained: age, sex, etiology of SBS, disease duration, gut anatomy (remaining small bowel length, colon in continuity), parenteral nutrition regime (volume and kcal per week), and treatment with teduglutide. Patients were routinely closely monitored for metabolic stability and stability of body weight as well as body composition during teduglutide treatment as described previously [16,21,24,25]. 

### 2.2. Citrulline Concentrations

Blood samples were collected at the outpatient department. Citrulline concentration was measured during clinical routine by high-performance liquid chromatography (HPLC); a plasma concentration of 40 (±10) µmol/L was used as the normal range [26,27]. 

### 2.3. Sequence Analysis of GLP-2 Receptor Gene

Whole blood samples for genetic analyses were collected, and sequence analysis was performed in 37 SBS patients and 3 healthy controls. All relevant regions (exon/intron boundaries, coding regions, and 5′UTR (5′ untranslated region) as putative promoter region of the GLP-2 receptor gene) were amplified. Specific primer designs according to the published sequences (GenBank NC_000017.11) were performed using the free software ExonPrimer (http://ihg.gsf.de/ihg/ExonPrimer.html (version primer 3 release 1.1.1, accessed on 17 April 2019) and according to the *GLP2R* promoter sequence and potential transcription factor binding sites as published [28]. DNA sequencing of the amplified regions was performed according to the manufacturer’s instructions (ABI Prism 310 genetic analyzer, Applied Biosystems, Waltham, MA, USA) and as previously reported [29].

Bioinformatic analyses for interpretation of the impact of identified genetic alterations to protein structure were performed using bioinformatic sites:

1. SIFT (sorting intolerant from tolerant) [30]

Based on sequence alignments and the specific physical features of each amino acid, the SIFT calculates a score between 0 and 1, reflecting the probable alteration in protein function caused by the amino acid change. A SIFT score ≤ 0.05 reflects a damaging amino acid substitution, and a score > 0.05 represents a tolerated amino acid substitution [30].

2. PolyPhen (Polymorphism Phenotyping) [31]

Based on physical properties, PolyPhen gives information about the impact of amino acid alterations on human protein structure and function. The PolyPhen score ranges from 0 to 1, and scores ≥0.85 are graded as probably damaging [31].

### 2.4. RNA Extraction, cDNA Synthesis, and Real-Time qRT-PCR

For RNA extraction from fresh intestinal biopsy samples, biopsies were collected in RNAprotect Tissue Reagent (#1017980, Qiagen, Hilden, Germany) immediately after tissue excision. RNA extraction was performed using the RNeasy Micro Kit (Qiagen #74004) according to the manufacturer’s protocol. The total RNA concentration and A260/A280 ratio were measured using a NanoQuant Infinite M200 Pro spectrophotometer (Tecan, Mennedorf, Switzerland). Samples with an A260/A280 ratio of less than 1.8 were excluded. For cDNA synthesis, the RT^2^First Strand Kit (Qiagen #330404) was used according to the manufacturer’s protocol. 

Quantitative real-time polymerase chain reaction (qRT-PCR) was performed using the FastStart Essential DNA Green Master mix (#06402712001, Roche, Basel, Switzerland) and QuantiTec Primer Assays™ (see below) or the RT^2^ Profiler™ PCR Array Human Tight Junctions (Qiagen #PAHS-143Z). We analyzed two different groups. Firstly, healthy controls (*n* = 7) compared to SBS patients without teduglutide treatment (*n* = 37) and secondly, a subgroup with paired data from SBS patients prior to initiation and while on teduglutide treatment for a minimum of 6 months (*n* = 18). Relative gene expression was calculated with the ΔCt method and given as 2^−ΔCt^ (∆Ct = Ct*GOI* − Ct*HKG)*).

For each assay, we used 1 µL cDNA in 20 µL PCR reactions; each sample was prepared twice. The thermal cycling parameters were 95 °C for 15 min, followed by 45 cycles of 95 °C for 60 s, 55 °C for 30 s, and 70 °C for 30 s. The cycle threshold (Ct) value of the gene of interest (GOI) was calculated with reference to the housekeeping genes (HKG) value. Relative gene expression was calculated with the ΔCt method and given as 2^−ΔCt^ (∆Ct = Ct*GOI* − Ct*HKG*).

The following Quantitec Primer Assays were used: sucrase-isomaltase (SI), Hs_SI_1_SG; Cytokeratin 20 (CK20), Hs_KRT20_1_SG; Marker of Proliferation Ki-67 (MKI67), Hs_MKI67_1_SG; sodium-dependent glucose transporter 1 (SGLT1), Hs_SLC5A1_1_SG; calcium/calmodulin-dependent serine protein kinase (CASK), Hs_CASK_1_SG; Tight junction protein 1 (TJP1/ZO-1), Hs_TJP1_1_SG; Occludin (OCLN), Hs_OCLN_1_SG; Claudin 10 (CLDN10), Hs_CLDN10_1_SG; Claudin 15 (CLDN15), Hs_CLDN15_1_SG; Crumbs cell polarity complex component 3 (CRB3), Hs_CRB3_1_SG. Housekeeping genes: beta-actin (ACTB), Hs_ACTB_1_SG; beta-2 microglobulin (B2M), Hs_B2M_1_SG; 40S ribosomal protein S9 (RSP9), Hs_RPS9_1_SG; Ribosomal protein lateral stalk subunit P0 (RPLP0), Hs_RPLP0_1_SG [32]. 

### 2.5. Immunohistochemistry

Small intestinal mucosal tissue sections (1–2 µm) were cut from paraffin blocks of archived patients’ endoscopically or surgically obtained samples (ZeBanC, Charité tissue bank, Berlin, Germany), dewaxed for standard histochemistry (H&E) and immunohistochemistry (IHC). For IHC, sections were dewaxed prior to heat-induced epitope retrieval. Sections were rinsed with running tap water and incubated with antibodies directed against Ki-67 (clone MIB1, Agilent, Santa Clara, CA, USA), SI (Abcam #224085), SGLT (Abcam #ab14685), CASK (clone K56A/50, Abcam, London, UK), or CK20 (clone D9Z1Z, Cell Signaling Technologies, Danvers, MA, USA) at room temperature. For the detection of CASK, the EnVision + Single Reagent (HRP. Mouse; Agilent) was used. For the detection of SI, SGLT, OCLN, and CK20, the EnVision+ Single Reagent (HRP. Rabbit; Agilent) was used. HRP was visualized with diaminobenzidine as chromogen (Agilent). The Dako REAL Detection System, Alkaline Phosphatase/RED, Rabbit/Mouse (Agilent), was used for the detection of Ki-67. Nuclei were counterstained with hematoxylin (Merck, Rahway, NJ, USA) and slides covered with glycerol gelatin (Merck). Primary antibodies were omitted in negative control sections. Stained sections were analyzed in a blinded fashion using an AxioImager Z1 microscope (Carl Zeiss Microscopy Deutschland GmbH, Oberkochen, Germany).

Relative epithelial cell numbers expressing Ki-67, as well as Ki-67 positive lamina propria mononuclear cells, were counted in five fields of vision. Fields were selected where crypts and villi were tangentially cut. One single investigator, blinded to the sample identification, performed the analyses.

### 2.6. Intestinal Permeability Measuring

Intestinal permeability was assessed using a triple carbohydrate absorption test, including sucrose, lactulose, and mannitol. The carbohydrate analytes were orally administered after overnight fasting, and urine was collected for 5 h. Collected urine was stored at −20 °C. For sample preparation, 500 µL of urine was mixed with 50 µL of internal standards (250 mM mesoerythritol, and 10 mM cellobiose). Then, 50 µL of 5-sulfosalicylic acid was added for protein precipitation, and Amberlite ion exchange resin (Amberlite IRN-150 Alfa Aesar, Haverhill, MA, USA) was added for desalting. Samples were mixed for 20 min, followed by centrifugation for 10 min at 13,000 rpm. A total of 10 µL of supernatant was diluted with 990 µL HPLC grade H_2_O. A total of 20 µL of diluted samples were injected for HPLC analysis. The recovery rate of each single carbohydrate in urine was measured by HPLC analysis (Shimadzu Nexera Bio, Kyoto, Japan; Operation and control by Labsolutions) using an electrochemical detector (Antec, Singapore, Decade Elite SCC). Chromatography was performed using a Dionex CarboPac PA1 IC guard column, a Dionex CarboPac PA1 IC separator as the main column (both Thermo Scientific, Waltham, MA, USA), and 125 mM NaOH (degassed) as eluent with a flow rate of 1 mL/min. The recovery rate of each carbohydrate was calculated using standard curves of sucrose, lactulose, and mannitol.

The lactulose/mannitol ratio was defined as the permeability index and was used as a marker for intestinal permeability (IP). The mannitol, lactulose and sucrose recovery rate were calculated as % of the ingested dose, and gastroduodenal permeability was calculated from the sucrose recovery rate. A mean value +2SD (standard deviation) of the control group defined the upper limit of normal IP (IP = 0.03), all as previously described [33,34].

### 2.7. Statistical Analysis

Statistical analyses were performed using GraphPad Prism for windows, version 10.0.1 (GraphPad Software, San Diego, CA, USA). Testing for association between study cohorts for citrulline, parenteral support, permeability testing results or gene expression results was performed by U Mann–Whitney or Wilcoxon test whenever appropriate. Correlation analysis was performed by non-parametric Spearman correlation analysis. Analyses for linear regression were performed using one-way ANOVA [35,36], the distribution of residues was analyzed by the D’Agostino–Pearson omnibus normality test and homoscedasticity was confirmed with the test for appropriate weighting [37,38]. *p*-values less than 0.05 were considered statistically significant.

## 3. Results

### 3.1. Study Cohort

A total of 59 SBS patients and 19 healthy controls agreed to participate in the study, and baseline characteristics are presented in Table 1. 

### 3.2. Parenteral Support

**Figure 1:**
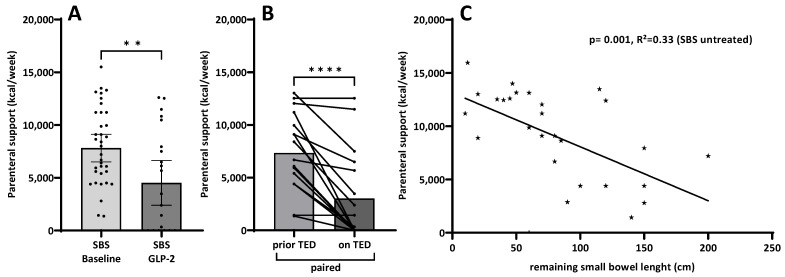
Analysis of parenteral support given as energy per week (kcal/week) in untreated and teduglutide-treated SBS patients and the relation with the remaining small bowel length. (**A**): Parenteral nutrition requirements were higher in untreated SBS patients compared to teduglutide (TED) treated patients (*n* = 36/24, ** *p* = 0.009 ^§^). (**B**): Paired analysis of SBS samples from couples prior to vs. while on teduglutide treatment (*n* = 17, **** *p* < 0.0001 ^#^). (**C**): Linear regression analysis of parenteral support (kcal/week) and remaining small bowel length (cm) in untreated SBS patients revealed an inverse relation (*n* = 36, *p* = 0.001, R^2^ = 0.33 ^$^). ^§^ Mann–Whitney, ^#^ Wilcoxon; ^$^ One-way ANOVA linear regression.

The degree of parenteral support is given as energy per week (kcal/week). The untreated SBS cohort was compared with the group of SBS patients on teduglutide treatment. Subgroup analysis was performed with paired data from SBS patients prior to initiation and while on teduglutide treatment for a minimum of 6 months. A significant reduction of parenteral calories was detected in both groups on teduglutide treatment (Figure 1A,B). Furthermore, as expected, a significant relation could be demonstrated between parenteral calories and remaining small bowel length in the untreated cohort (Figure 1C). 

### 3.3. Citrulline 

**Figure 2:**
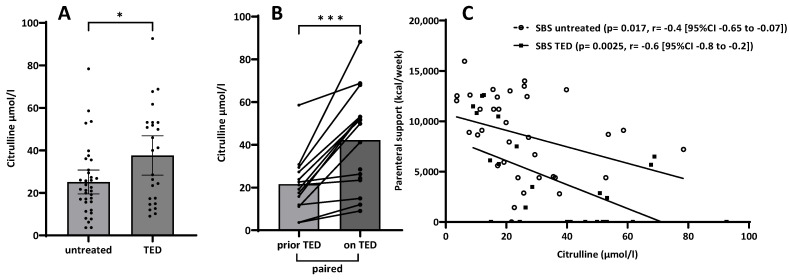
Analysis of Citrulline plasma levels (µmol/L) in untreated and teduglutide-treated SBS patients and the correlation with parenteral support. (**A**): The untreated SBS cohort showed lower citrulline levels compared to teduglutide (TED) treated patients (*n* = 36/24, * *p* = 0.045 ^§^). (**B**): Paired analysis of SBS samples from couples prior to vs. while on teduglutide treatment (*n* = 14, *** *p* < 0.001 ^#^). (**C**): An inverse correlation of parenteral support (kcal/week) with citrulline in plasma was found for both untreated SBS patients (*n* = 36, *p* = 0.017, r = −0.4 [−0.65 to −0.07] ^&^) and SBS patients while on teduglutide treatment (*n* = 24, *p* = 0.0025, r = −0.6 [−0.8 to −0.2] ^&^). ^§^ Mann–Whitney, ^#^ Wilcoxon; ^&^ Spearman correlation coefficient with 95% confidence level.

As expected, citrulline plasma levels in SBS patients were reduced when compared to the normal range; however, significantly higher (on average, almost normal) citrulline values were seen in the group with teduglutide treatment (Figure 2A). Furthermore, the paired data set analysis in patients prior to and while on teduglutide treatment showed a strong increase in citrulline values while on teduglutide treatment (Figure 2B). 

In addition, correlation analysis revealed a significant inverse correlation for higher citrulline values and lower caloric parenteral requirements in both the untreated SBS group as well as for patients on teduglutide treatment (Figure 2C). 

### 3.4. Sequence Analysis of GLP-2 Receptor Gene

Variants in the GLP-2 receptor gene might contribute to the response to both endogenous as well as exogenous GLP-2 and its analogs, and, therefore, the GLP-2 receptor gene sequence was analyzed in detail in thirty-seven SBS patients and three healthy controls. Table 2 shows identified sequence variants of the GLP-2 receptor gene. Three variants were identified with nonsynonymous amino acid changes. The p.R41S variant in *GLP2R* is located in the N-terminal end close to the hormone-binding domain. Potentially, this can change protein structure or function. In contrast, the SIFT score for this mutation is 0.04, and the PolyPhen score is 0.00. The reported MAF (minor allele frequency) is 0.0006. This variant occurs solely in one SBS subject and not in controls. Whether this variant can influence GLP-2 receptor functions has to be further elucidated. 

The p.V234I variant in *GLP2R* is located in transmembrane domain 2, and the variant p.D470N is located in the C-terminal end. Both prediction tools classified these variants to be benign. The p.V243I variant was only found once in a SBS patient and not in controls. The reported MAF is 0.003. It is not likely to be a variant with changes in protein function but has to be further analyzed. The p.D470N is a common single nucleotide polymorphism (SNP) with a reported MAF of 0.27. In our cohort, the detected MAF was 0.25, and the SNP occurs in SBS patients as well as in healthy subjects. This variant is unlikely to cause alterations in protein function. 

Further, we identified the SNPs p.G158G, p.H504H, IVS5-24g<, IVS8+15a<g, IVS9-7t<g, IVS11+22a<g in SBS patients as well as in controls. All these variants are synonymous amino acid exchanges or intronic variants with no putative effect on protein function or splice sites. In addition, we analyzed an 800 bp sequence in the 5′UTR upstream of the transcription start site (ATG) in exon 1 of the *GLP2R* gene. The variant c.1-199 G<A lies in a region previously identified as a potential SP1 transcription factor binding site. The variants c.1-608 G<A, c.1-635 C<G, and c.1700 C<G did not affect reported potential transcription factor binding sites. The three variants c.1-199 G<A, c.1-608 G<A, and c.1-770 C<G occurred with the same MAF and were found in the same subjects. It is likely that these variants are linked to the same allele. The variant c.1-635 C<G is only detected in one SBS patient and not reported in the dSNP database. Whether the identified variants in the 5′UTR region of the GLP-2 receptor gene affect GLP-2 receptor protein expression should be further elucidated. 

### 3.5. qPCR Expression Profiles of Tight Junction Genes and Epithelial Markers

In the first step, we analyzed gene expression in mucosal biopsies from nine SBS patients and four healthy subjects by applying a commercially available RT-PCR profiler Array (Qiagen) for a panel of 82 different tight junction genes. A large majority of analyzed genes in this panel did not show relevant differences in gene expression neither between healthy controls and any SBS patients nor between SBS couples prior to treatment compared to while on teduglutide treatment with the exception of a few genes, which gave first hints for gene expression differences between subgroups. Therefore, an extended analysis in a larger patient cohort was performed, including a few genes from the tight junction panel and adding some typical intestinal epithelial marker genes: *CLDN10, CLDN15, OCLN, ZO-1, CRB3, CASK, SI, SGLT1, MKI67,* and *CK20* (Table 3). 

### 3.6. Immunohistochemistry

To further study the potential role of the genes whose expression had been analyzed by qPCR, protein expression was assessed immunohistochemically (IHC) with small intestinal tissues from the same patients under comparable conditions without additional pathology beyond SBS. 

**Figure 3:**
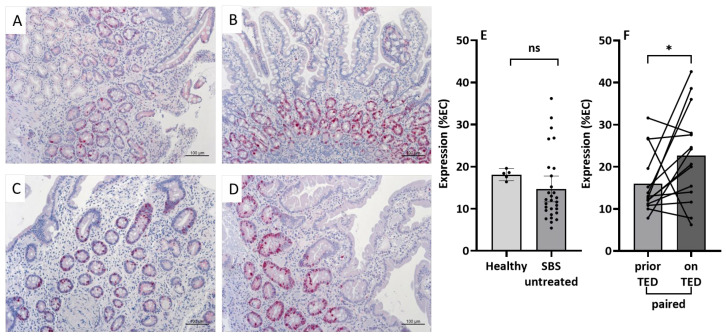
Immune staining for Ki-67 in small intestinal biopsies of SBS patients prior to (**A**,**C**) and with teduglutide (TED) treatment (**B**,**D**). Bars 100 µm. Ki-67 expression is restricted to the nuclei of crypt cells. With teduglutide treatment, Ki-67 protein expression increased. (**E**,**F**): Quantification of immune staining for Ki-67. Data were given as percentage of Ki-67 expressing enterocytes (% EC). (**E**): SBS patients without TED showed less Ki-67 expressing crypt cells compared to healthy controls (*n* = 29/5, *p* = 0.083 ^§^). (**F**): The paired analyses of SBS couples prior and while on TED treatment showed more Ki-67 expressing crypt cells when patients were treated with TED (*n* = 14, * *p* = 0.04 ^#^). ^§^ Mann–Whitney, ^#^ Wilcoxon, ns, not significant.

Figure 3 shows immune staining results from small intestinal biopsies for the proliferation marker Ki-67 in two SBS patients without (Figure 3A,C) and with teduglutide treatment (Figure 3B,D). The positive immune staining is restricted to the nucleus and is, as expected, only present in the crypts. In SBS patients with teduglutide treatment, the Ki-67-positive immune staining was increased, as indicated by the percentage of Ki-67-positive epithelial cells (EC) (Figure 3B,D). Figure 3E,F represents the quantification results of immune staining for Ki-67. No significant differences in Ki-67 immune staining were observed in SBS patients without teduglutide compared to healthy controls (Figure 3E). In contrast, Ki-67-positive immune staining increased when SBS patients were treated with teduglutide (Figure 3F).

**Figure 4:**
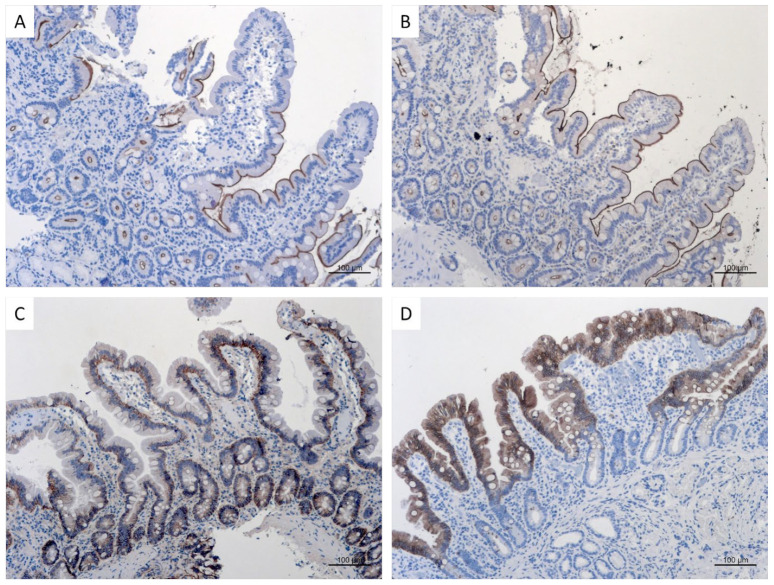
Representative immune staining results for corresponding proteins of small intestinal biopsies from SBS patients prior to teduglutide treatment. (**A**): sucrase-isomaltase (SI), and (**B**): Sodium-dependent glucose transporter 1 (SGLT-1). Both proteins are brush border proteins consistent with the staining at the outer edge of enterocytes. For SI, a higher expression in the middle and lower part of the villi was seen. (**C**): Calcium/calmodulin-dependent serine protein kinase (CASK). CASK is suggested to function as a cytoskeletal membrane scaffold that coordinates signal transduction pathways within the cytoskeleton. The staining for CASK is located in the lateral and mainly basolateral membrane. (**D**): Cytokeratin 20 (CK20). Cytokeratin 20 belongs to intermediate filament proteins responsible for the cytoskeletal structural integrity of epithelial cells and is thus mainly expressed in the cytoplasm of epithelial cells of the small and large intestines. CK20 is used as a marker for epithelial differentiation; therefore, the staining is less prominent in the crypts and more intense towards the villi. Bars 100 µm.

To corroborate the results from gene expression analyses, we performed immune staining for sucrase-isomaltase (SI) (Figure 4A) and the sodium-dependent glucose transporter 1 (SGLT-1) (Figure 4B). Both proteins are brush border proteins consistent with the staining at the outer edge of enterocytes. For SI, a higher expression in the middle and lower part of the villi was reported [39] and confirmed in our cohort. Due to the localization of the proteins at the outer edge of enterocytes, a reliable quantification within each respective villus was not possible. 

A representative example of baseline IHC results for calcium/calmodulin-dependent serine protein kinase (CASK) is shown in Figure 4C. For CASK, it is suggested that it may function as a cytoskeletal membrane scaffold that coordinates signal transduction pathways within the cytoskeleton. The staining for CASK was in concordance with the literature located on the lateral and mainly basolateral membrane [40]. Figure 4D shows the results for a typical enterocyte marker cytokeratin 20 immune staining. In general, keratins are intermediate filament proteins responsible for the cytoskeletal structural integrity of epithelial cells and thus mainly expressed in the cytoplasm of epithelial cells of the small and large intestine. 

### 3.7. Intestinal Permeability Testing 

#### 3.7.1. Intestinal Permeability in Short Bowel Syndrome

**Figure 5:**
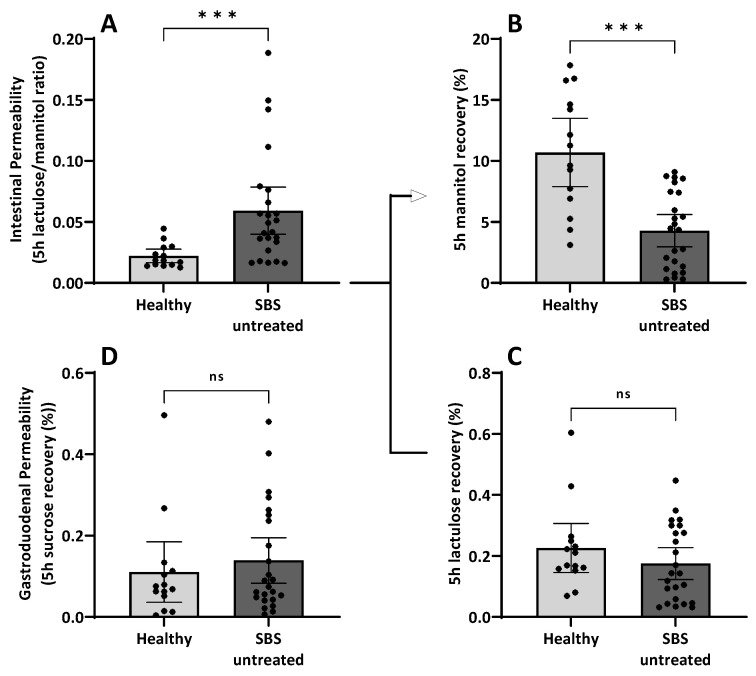
Analysis of intestinal permeability as carbohydrate recovery rate in untreated SBS patients and healthy controls in urine 5 h after oral carbohydrate ingestion. (**A**): SBS patients showed an increased intestinal permeability index expressed as lactulose/mannitol ratio compared to healthy controls (*p* < 0.001 ^§^). (**B**): The mannitol recovery rate was decreased in SBS patients (mean SBS 4.3% vs. healthy 10.7%, *p* < 0.001 ^§^), whereas the lactulose recovery rate (**C**) was similar to healthy subjects (mean SBS 0.18% vs. healthy 0.22%, *p* = 0.32 ^§^). Therefore, the elevated lactulose/mannitol ratio is caused by the reduced mannitol recovery rate rather than the lactulose recovery rate, not indicating increased paracellular leakage but rather decreased transcellular transport due to decreased epithelial enterocyte cell mass. (**D**): Gastroduodenal permeability measured as sucrose recovery rate was similar to healthy subjects (^§^ Mann–Whitney, *** *p*-value < 0.001, ns, not significant). Please recognize the unusual order of panels (**C**,**D**) in the lower part of this figure.

Intestinal permeability (IP) testing was performed in SBS patients without teduglutide treatment and healthy controls. SBS patients showed an increased lactulose/mannitol ratio compared to healthy controls. However, regarding the single carbohydrate recovery rate, the mannitol recovery was decreased in SBS patients (mean SBS 4.3% vs. healthy 10.7%), whereas the lactulose recovery rate was similar to healthy subjects (mean SBS 0.18% vs. healthy 0.22%). Therefore, the elevated lactulose/mannitol ratio is caused by the reduced mannitol recovery rate rather than the lactulose recovery rate, not indicating increased paracellular leakage but rather decreased transcellular transport due to presumed lower enterocyte cell mass. No differences were found for the sucrose recovery rate (a marker for gastroduodenal permeability) (mean SBS 0.14% vs. healthy 0.11%) (Figure 5A–D). 

#### 3.7.2. Linear Regression of Intestinal Permeability Testing with Remaining Small Bowel Length

**Figure 6:**
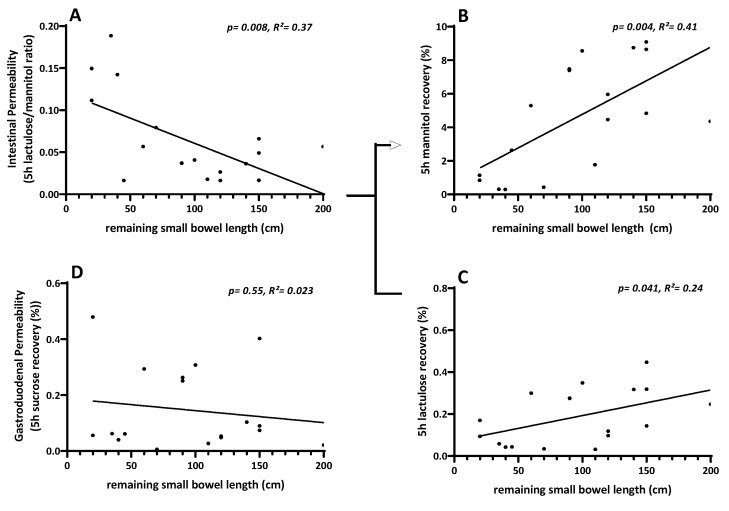
Linear regression of intestinal permeability as sugar recovery rates in SBS patients with the remaining small bowel length. (**A**): The lactulose/mannitol ratio showed a strong negative relation with the remaining small bowel length (*p* = 0.008, R^2^ = 0.37 ^$^). In contrast, for the mannitol recovery rate (**B**), a strong positive relation with the remaining small bowel length was found (*p* = 0.004, R^2^ = 0.41 ^$^), while lactulose recovery (**C**) showed less correlation (*p* = 0.041, R^2^ = 0.24 ^$^). No relation was found for sucrose recovery (**D**) (*p* = 0.55, R^2^ = 0.023 ^$^). ^$^ One-way ANOVA linear regression. Please recognize the unusual order of panels (**C**,**D**) in the lower part of Figure 6.

Carbohydrate recovery rates were further analyzed by linear regression with remaining small bowel length (rSBL) in SBS patients. The lactulose/mannitol ratio showed a strong negative relationship with the remaining small bowel length. In contrast, for the mannitol recovery rate, a strong positive relation with the remaining small bowel length was found, while lactulose recovery showed less correlation. No relationship was found for sucrose recovery (Figure 6A–D). This is in concordance with the interpretation of reduced mannitol recovery being most importantly influenced by the relatively reduced enterocyte cell mass in SBS patients and being the driver of the relatively increased lactulose/mannitol ratio, which is usually considered a marker of intestinal permeability in the anatomically unaltered small intestine.

#### 3.7.3. Intestinal Permeability Testing and Treatment with Teduglutide

**Figure 7:**
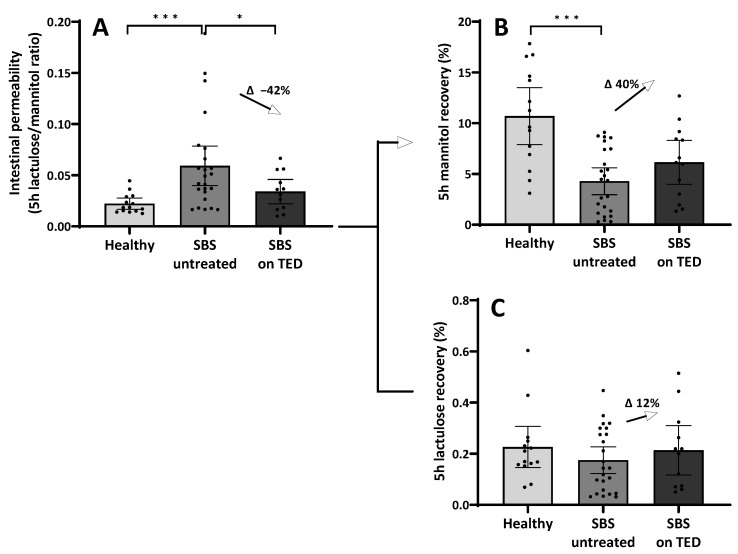
Analysis of intestinal permeability in untreated SBS patients or on teduglutide (TED) treatment. (**A**): SBS patients on teduglutide treatment showed a significantly lower lactulose/mannitol ratio (mean 0.03). The reduction was about 42% compared to the mean value in the untreated group (mean 0.06, * *p* = 0.04 ^§^). (**B**): Mannitol recovery was elevated in patients treated with teduglutide as compared to untreated patients (without TED 4.3%, with TED 6.7%; Δ mannitol recovery 40%). (**C**): Lactulose showed only minor changes (without TED 0.18%, with TED 0.2%; Δ lactulose recovery 12%). For comparison, mean baseline levels of healthy controls were added to each graph (please refer to Figure 5, *** *p*-value < 0.001). ^§^ Mann–Whitney.

Results of intestinal permeability testing were compared between untreated SBS patients (*n* = 24) and SBS patients with teduglutide treatment (*n* = 13). The group of SBS patients with teduglutide treatment showed a significantly lower lactulose/mannitol ratio. The reduction was about 42% compared to the mean value in the untreated group. In contrast, mannitol recovery was elevated in patients treated with teduglutide compared to untreated patients (without TED 4.3%, with TED 6.7%; Δ mannitol recovery 40%). Lactulose showed only minor changes (without TED 0.18%, with TED 0.2%; Δ lactulose recovery 12%). Sucrose recovery showed no changes (Figure 7A–C).

#### 3.7.4. Correlation of Intestinal Permeability Testing and Parenteral Support

**Figure 8:**
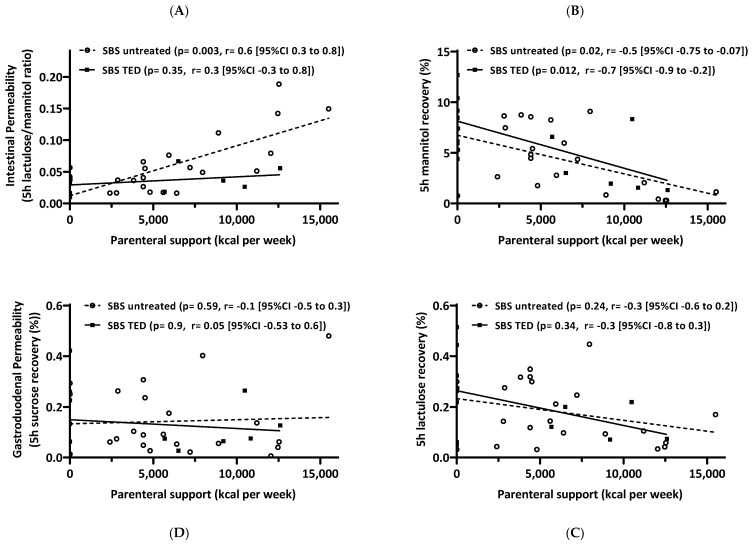
Correlations between intestinal permeability results and caloric parenteral support (kcal/week) in SBS patients without or with teduglutide (TED) treatment. (**A**): A strong positive correlation was found for the lactulose/mannitol ratio in the group of untreated SBS patients (*p* = 0.003, r = 0.6 (0.3 to 0.8) ^&^). This correlation was not present in the group of SBS patients with teduglutide treatment (*p* = 0.35, r = 0.3 (−0.3 to 0.8) ^&^). (**B**): An inverse correlation was found for mannitol recovery and parenteral support for both groups (untreated SBS *p* = 0.02, r = −0.5 (−0.75 to −0.07) ^&^, SBS on TED *p* = 0.012, r = −0.7 (−0.9 to −0.2) ^&^). No relation was found for lactulose recovery (**C**) or sucrose recovery (**D**). ^&^ Spearman correlation coefficient with 95% confidence level. Please recognize the unusual order of panels (**C**,**D**) in the lower part of Figure 8.

Finally, the results of IP testing were analyzed for correlation with caloric parenteral support (kcal/week). A strong positive correlation was found for the lactulose/mannitol ratio in the group of untreated SBS patients. This correlation was not present in the group of SBS patients with teduglutide treatment. An inverse correlation was found for mannitol recovery and parenteral support for both groups. No relation was found for lactulose recovery or sucrose recovery (Figure 8A–D). This suggests that classical intestinal permeability testing by oral carbohydrate ingestions and 5 h urinary recovery in SBS patients is, in fact, a surrogate test for epithelial enterocyte mass and (probably transcellular) transport function for mannitol rather than paracellular permeability for lactulose, which was only minorly changed and did not significantly correlate with parenteral nutrient requirements.

## 4. Discussion

### 4.1. Summary of Results

Biomarkers for intestinal function in human chronic intestinal failure due to SBS are scarce but are needed for tailoring both nutritional and rehabilitative treatment to individual nutritional needs and intestinal capacity in these patients. In the literature, a decrease in intestinal permeability has been reported in the context of GLP-2 actions [41]. However, the actions of GLP-2 are mediated through the GLP-2 receptor, although the complete downstream mechanism has not yet been fully understood. Clinically, patients respond to GLP-2 treatment with considerable variability. Having this in mind, a multifaceted approach was taken in a search for potential either genetic, molecular, structural or functional biomarkers from samples of patients with SBS obtained during routine clinical management, including blood samples, small intestinal biopsies and 5 h urinary samples from an oral carbohydrate ingestion test. These were correlated also with standard clinical information on parenteral nutrition and remaining small bowel anatomy (Table 1; Figure 1 and Figure 2). 

While analyses for GLP-2 receptor polymorphisms as a potential influencer of response to teduglutide treatment did not reveal relevant signals (Table 2), gene expression analyses by qPCR of genes potentially related to small intestinal epithelial characteristics did unexpectedly not show any associations with epithelial tight junction genes but rather with epithelial transport-associated molecules such as SI and SGLT-1 (Table 3). Therefore, the reduction of enterocyte mass results in the need for parenteral compensation in correlation with the corresponding remaining small bowel length (rSBL) (Figure 1C), which is also associated with the only clinically available biomarker citrulline (Figure 2C). 

However, intestinal rehabilitative treatment of SBS patients with the GLP-2 analog teduglutide, which is known to enhance small intestinal absorption as well as morphology with increased both villus length and crypt depth [25,42], is able to decrease parenteral calorie requirements (besides volume requirements as has been shown in numerous studies [24,43,44]). This could be shown in grouped cohort comparisons as well as in a paired subcohort (Figure 1A,B). The decrease in parenteral calorie requirements is paralleled by an inverse increase in citrulline serum concentrations (Figure 2A,B), indicating increased enterocyte mass, as has been shown previously [22,25]. In this study, it is suggested that not only a teduglutide-induced increase in enterocyte mass per se, as demonstrated by increased Ki-67-index in teduglutide-treated patients, may contribute to this (Figure 3). Additionally, the increased expression of nutrient transport-associated genes such as *SI* and *SGLT-1* (Table 3) very likely play an important role, which is paralleled by intense evidence of protein expression in the small intestinal epithelium (Figure 4).

Finally, while applying a classical oral carbohydrate ingestion and urinary recovery “intestinal permeability” (IP) test, it was discovered that in SBS patients, IP, as assessed by lactulose/mannitol ratio, is formally “increased” (Figure 5A) as has been described earlier [8,9]. However, by analyzing the actual recovery and thus transport data themselves, it turned out that paracellular lactulose transport was not altered at all (Figure 5C), as should be expected if increased “leakiness” of the small intestine played a pathophysiological role. Rather, decreased transcellular mannitol transport (Figure 5B), as can be expected with reduced enterocyte mass, was observed in SBS, while gastroduodenal transport was shown to be not different in SBS as compared to healthy controls (Figure 5D). This observation of mannitol-uptake as a “functional biomarker” of enterocyte mass also strongly correlated with rSBL (Figure 6). This indicates the specific role of enterocyte mass and, more importantly, function for nutrient uptake as had been suggested by gene and protein expression in the earlier investigation (Table 3 and Figure 4) as well as by citrulline serum concentrations and its correlation with rSBL (Figure 2). Even more importantly, teduglutide treatment of parenteral nutrition-dependent SBS patients seemed to formally reverse “intestinal permeability” (Figure 7), but this observation was again caused by increased mannitol recovery and thus uptake rather than paracellular lactulose uptake, indicating the role of increased enterocyte mass through treatment with teduglutide in these patients. Quite concordantly, these effects also correlated with rSBL significantly only for mannitol recovery but neither for lactulose or sucrose recovery (Figure 8). 

### 4.2. Parenteral Support as a Clinical Surrogate for Small Intestinal Function

To compensate for nutrient and fluid deficiency, SBS patients are supported with parenteral nutrition and intravenous fluids. The need for parenteral support strongly reduces quality of life and harbors, among others, the risk of life-threatening catheter infections [16]. One of the main therapeutic strategies in SBS is to reduce the volume and/or days of parenteral support in order to improve the patient’s quality of life. A reduction of >20% in parenteral support volume from baseline has been classified as a response to GLP-2 analog treatment [21]. In this study, we could confirm (Figure 1) previous findings with teduglutide treatment reducing requirements by volume and as calorie demands per week [25]. Furthermore, we could show a significant inverse correlation of parenteral support with the remaining small bowel length, which is in concordance with analyses from phase III trials as well as real-world data [44,45]. However, although data on parenteral support are readily available in each patient, due to disease heterogeneity, parenteral support is complex and quite variable [15,21,25,43], which limits the use of parenteral support as a clinical surrogate marker of small intestinal function.

### 4.3. Citrulline as a Biomarker

Another clinically available biomarker is the blood plasma concentration L-citrulline [22,25,26,46]. L-Citrulline is a non-essential amino acid and is known to be synthesized from precursor amino acids like glutamine in enterocytes of the proximal small intestine (mostly duodenum and jejunum) [47,48,49,50]. 

A first study by Crenn et al. revealed reduced citrulline concentrations in patients with small bowel syndrome [22]. In addition, it could be shown that citrulline is a marker of enterocyte mass in patients with villus atrophy without intestinal resections [26]. Furthermore, citrulline plasma concentrations are suggested for use as a prognostic marker for parenteral nutrition weaning (if citrullinemia is >20 micromol/L) [47], and increased plasma concentrations of citrulline were observed in SBS patients when treated with the GLP-2 analog teduglutide [21]. In concordance with these, we report a reduced citrulline concentration in SBS patients compared to healthy subjects and a significant correlation of citrulline plasma levels with the parenteral support (Figure 2). The use and interpretation of citrulline values are, however, controversially discussed in the literature. A study by Picot et al. concluded that citrulline is less of a marker of overall intestinal mass than for absorptive small bowel function [51]. In contrast, a meta-analysis of citrulline with gut function by Fragkos et al. revealed that citrulline appears to be a strong marker of enterocyte mass, while its correlation with intestinal absorption seemed weaker [52]. Since, in addition, citrulline levels are influenced by age and renal failure, it is a poor biomarker in these frequently abundant conditions in SBS patients [49,53,54,55]. Finally, the use of citrulline as a biomarker is also limited by availability and complex measuring techniques, not readily available to many clinicians and their patients [16]. Thus, citrulline has proven to be a somewhat useful marker for enterocyte mass; however, values have to be interpreted with caution, and future studies should look into the correlation between citrulline and other potential biomarkers, such as intestinal permeability.

### 4.4. GLP-2 Receptor Gene Analysis

As a mediator of GLP-2 action, the GLP-2 receptor gene seems of interest in SBS because it is directly involved in GLP-2 recognition and thus potentially influential on intestinal rehabilitation, be it spontaneous or pharmacologically enhanced. Here, to our knowledge, for the first time, we analyzed the GLP-2 receptor gene (*GLP2R*) and the putative promoter region for sequence variants with potential alterations in protein function, which in turn could result in varying teduglutide treatment responses in a subset of patients (Table 2). The sequence analyses of the GLP-2 receptor gene revealed three variants with consequences to the amino acid sequence. Two of them are very rare variants and one is a common sequence variant. Bioinformatic prediction tools did not estimate changes in protein function. Therefore, it is unlikely that one of these variants influences the treatment response to teduglutide [11,56]. 

### 4.5. Gene Expression Analysis of Multiple Epithelial Small Intestinal Genes

Beyond genes themselves and their structure, gene expression is an important regulator of organ function. Therefore, gene expression analyses from mucosal biopsies for tight junction genes and epithelial markers were performed in healthy controls, untreated SBS patients and SBS patients treated with teduglutide by qPCR (Table 3); in 18 patients, tissue samples were available for both clinical situations (prior and on teduglutide treatment). The analyses of a panel of 82 tight junction-related genes revealed that the large majority of tight junction-associated genes did not show different expression levels between healthy controls and SBS patients or SBS patients before and with teduglutide treatment. This is, in fact, in concordance with the later discussed evidence of largely unaltered paracellular transport in both teduglutide-naïve and teduglutide-treated SBS patients.

Therefore, in an extended analysis, potentially relevant gene expression beyond the extensive tight junction panel was studied in a larger cohort, adding typical epithelial marker genes. 

Sucrase-isomaltase (SI) is an enterocyte-specific, membrane-bound brush-border enzyme exclusively expressed in the small intestine [57]. By qPCR, SI demonstrated reduced gene expression in untreated SBS patients compared to healthy controls (Table 3). In contrast, SI gene expression increased in SBS patients treated with teduglutide compared to untreated SBS patients, and the correct protein localization could be confirmed by IHC (Figure 4A). Changes in SI gene expression are a regulatory step in enzyme activity [39]; for example, mouse models have shown a regulation of SI expression by a carbohydrate diet [58]. Therefore, the reduced SI expression in untreated SBS patients might be explained by decreased luminal availability of carbohydrates as a result of reduced oral intake and accelerated intestinal passage. On the other hand, with teduglutide treatment, intestinal passage decelerates, chyme thickens, and thus, the availability of luminal carbohydrates increases, which may lead to increased SI expression. Furthermore, in a study performed in a total parenteral nutrition (TPN) feed rat model, it could be shown that an increase in mRNA expression was induced by GLP-2 administration and not by the TPN itself [59]. In addition, a study in neonatal piglets revealed GLP-2 stimulated jejunal SI mRNA abundance [60]. Thus, our observations in SBS patients are concordant with results with SI in animal models, and SI could be a good candidate as a mostly scientific biomarker in small intestinal biopsies from SBS patients.

Sodium/glucose cotransporter 1 (SGLT1) is a human protein encoded by the *SLC5A1* gene and is mainly expressed in the upper regions of the small intestine [61,62,63]. While expression of SGLT1 was reduced in SBS patients compared to healthy controls, it was considerably increased in SBS patients treated with teduglutide compared to treatment-naïve SBS patients (Table 3) and its characteristic localization was confirmed by IHC (Figure 4B). In an initial study in mice treated with native GLP-2, a higher protein activity compared to untreated mice was found, but authors could not find elevated mRNA levels estimated by Northern blot analysis [64]. In contrast, in an vivo GLP-2 infusion model in rats, authors showed GLP-2 infusion not only increased the transport capacity of the glucose transporter but also the incorporated SGLT1 protein amount [65]. Differences between studies may be explained by different species, detection methods, and the finding that SGLT1 mRNA expression is higher in humans than in rats or mice [66]. Thus, SGLT1 is obviously a transporter molecule regulated by GLP-2 and its analogs in SBS patients. However, since quantitation in biopsies is difficult, and tissue qPCR is usually not possible on routine biopsies, it is a physiologically interesting molecule but probably has limited potential as a clinical biomarker of small intestinal function. 

The calcium-calmodulin serine protein kinase (*CASK*) gene encodes a protein kinase belonging to the membrane-associated guanylate kinase (MAGUK) family and is the mammalian orthologue of LIN2, a *Caenorhabditis elegans* MAGUK required for basolateral localization of the epidermal growth factor receptor (EGFR) in certain polarized epithelial cells [67]. CASK is ubiquitously expressed, and its interaction with LIN7, another MAGUK, is evolutionarily conserved. In this study, CASK demonstrated a remarkable reduction in gene expression in SBS patients compared to healthy controls and an increased gene expression in SBS patients treated with teduglutide (Table 3) and its characteristic protein localization was confirmed by IHC (Figure 4C). However, the exact function of intestinal CASK is not well understood. It is supposed to play a role in transmembrane protein anchoring, especially on the lateral and basolateral membrane and ion channel trafficking [68]. However, a number of gut growth factors have been implicated as important modulators for adaptive response or as direct mediators of the action of GLP-2, such as the epidermal growth factor (EGF) and insulin-like growth factor-1 (IGF-1) [69,70]. The ErbB-2 signaling pathway has been identified as an essential component of the signaling network regulating the adaptive mucosal response to refeeding in the mouse intestine [71]. Therefore, the observation of this study suggests that gene expression changes of CASK may be related to changes in growth factor expression during both spontaneous intestinal adaptation and teduglutide-stimulated intestinal rehabilitation, but this hypothesis has to be further elucidated. Although potentially linked to the presumed mode of action of GLP-2, CASK is, therefore, currently not a likely candidate as a routine biomarker for small intestinal function in SBS patients. 

The Ki-67 protein is well known as a cellular marker for proliferation [72,73]. In our study, we could not reveal any relevant changes in gene expression for Ki-67 in our analyzed groups when normalized to housekeeping genes in qPCR (Table 3). Instead, by semi-quantitative immune staining, we could detect relevant differences in protein expression. A reduced percentage of protein expressing enterocytes was found in SBS patients as compared to healthy controls. In contrast, the percentage of Ki-67 expressing cells increased in teduglutide-treated tissue samples compared to values prior to treatment (Figure 3). The apparent discrepancies between the results of the two methods could be due to additional Ki-67 protein expression in the lamina propria, which can falsify qPCR results. In addition, qPCR results will be influenced by the crypt villi ratio in tissue biopsies. The positive impact of GLP-2 on proliferation has been shown in many studies [10,41,74,75], while the information on Ki-67 as a proliferation marker in SBS patients is limited. In a case report for a pediatric SBS patient, no apparent changes in positive Ki-67 cells were seen during GLP-2 treatment [76]. In contrast, another study analyzing the effects of GLP-2 on the proliferation and apoptosis of intestinal epithelial cells in SBS rat models revealed an elevated percentage of Ki-67 expressing cells [77]. Thus, the results of this study suggest that spontaneous intestinal adaptation (concurrent with parenteral nutrition, reduced oral nutrient intake, and possibly reduced endogenous GLP-2 levels due to extensive resection) may result in decreased enterocyte proliferation, which can be reversed and even exceeded by teduglutide treatment. As a biomarker, Ki-67 determination relies on small intestinal biopsies, but it could be determined in routine pathology samples and thus be an interesting response indicator for treatment with GLP-2 analogs. 

Cytokeratin 20, an intermediate filament protein mainly expressed in the cytoplasm of epithelial cells of the small and large intestine [78]. It has been shown to be highly expressed in highly differentiated cells and is used as a marker for carcinomas of different epithelial origin [77,78,79,80,81,82,83]. In this analysis, we confirmed proper protein distribution in intestinal epithelia (Figure 4C), but no relevant changes were found in gene expression (Table 3), suggesting that cellular differentiation of enterocytes in SBS patients was appropriate according to CK20-expression under both conditions, teduglutide-naïve and treated SBS patients. The use as a biomarker beyond maintained intermediate filament expression in biopsies of SBS seems, therefore, unlikely. 

No relevant changes in gene expression could be detected for the tight junction proteins occludin (*OCLN*), zonula occludens protein (*ZO-1*), claudin 10, and 15 (*CLDN10, -15*), and Crumbs cell polarity complex component 3 (*CRB3*) (Table 3). This corresponds to the findings of Reiner et al. [84], who used a mouse SBS model to study the tight junction leak pathway. The expression of tight junction genes *OCLN*, *ZO-1*, and *ZO-2* were analyzed, but their expression levels were stable in the SBS situation and were not influenced by teduglutide. Therefore, direct study of components of tight junctions has not been shown to be substantially disturbed by small intestinal resection or response to it, i.e., intestinal adaptation. The analyzed tight junction molecules, therefore, seem neither to be substantially involved in the pathophysiology nor to be candidates as biomarkers in SBS patients.

### 4.6. Functional Assessment of Intestinal Barrier by Intestinal Permeability Testing

Intestinal barrier function is finely regulated to balance between protection against pathogens and facilitate the absorption of nutrients and fluids [85]. The term intestinal permeability is often used imprecisely in the context of a so-called “leaky gut” suggesting the passage of harmful substances such as toxins and bacteria through the intestinal epithelium into the underlying tissue. While larger molecules can only pass the epithelium through the paracellular route, which is highly regulated by junctional complexes between the cells, smaller molecules can also pass along the transcellular route either via passive flux through membranes, aqueous pores, endocytosis, or even active carrier-mediated absorption [86]. Different in vitro or in vivo methods were used for intestinal permeability testing. Transepithelial electrical resistance (TEER) measures ionic conductance of the paracellular pathway in the epithelial monolayer, while mounted tissue can be studied in Ussing chamber experiments [87,88,89,90,91,92]. Other in vitro methods determine the flow of biomolecules of different sizes from the apical to the luminal side of the cell monolayers or mounted tissue [93,94]. All of these methods gave valuable information; however, this information only describes the paracellular route [95,96]. 

For the study of epithelial transport, oral gavage of biomolecules (e.g., carbohydrates, fluorescently labeled dextrans, polyethylen glycol, or 51Cr-EDTA) followed by detection of the recovery rate in blood or urine are commonly used methods in animals [94,97,98]. Administration of harmless and inert carbohydrates is used for humans [99,100], where the most common assay is the measurement of lactulose and mannitol and the calculation of the lactulose/mannitol ratio as permeability index [101,102]. Studies by Bijlsma et al. and Delahunty et al. revealed strong differences among species in mannitol recovery and further reduced lactulose/mannitol ratios. Differences in villus heights, which are much higher in humans as compared to rodents, may at least in part explain this [103,104]. In vitro, a high mannitol recovery rate gets lost with the absence of blood flow because the antidromic blood flow in villus vessels maintains hyperosmolality at the villus tips [103,105,106].

In this study, we used defined oral ingestion of sucrose, lactulose and mannitol and measured urinary excretion to estimate intestinal permeability [29,107]. Lactulose recovery reflects the paracellular transport and is typically increased in patients with a leaky gut syndrome, while mannitol recovery is used as a marker for the transcellular pathway and is rather attributed to the gut length or enterocyte mass [108]. We found high lactulose/mannitol ratios in SBS patients (Figure 5A), but this elevated ratio was caused by a reduced mannitol recovery (Figure 5B) rather than a higher lactulose recovery (Figure 5C) as typically seen in inflammatory diseases [102,109]. Lactulose recovery in the SBS cohort was instead comparable to heathy controls and gave no signals for impairment of the paracellular route. This was further supported by our findings for sucrose recovery, which serves as a marker for gastroduodenal permeability since it is immediately cleaved by disaccharidases upon entering the jejunum [33]. Since the stomach or duodenum were not resected, the recovery rate of sucrose was comparable to healthy controls (Figure 5D). For comparison, a true “leaky gut” situation is often associated with an increased sucrose recovery [33,87], which is not the case in SBS patients. In contrast, in teduglutide-treated SBS patients, we found elevated levels of mannitol recovery compared to untreated SBS patients (Figure 7), while we could not find significant changes for lactulose or sucrose recovery. These findings are supported by a placebo-controlled crossover study, where a small cohort of eight SBS patients were analyzed for intestinal permeability (lactulose/mannitol ratio) prior to and after 7 days of teduglutide. In concordance with our results, they observed an increase in mannitol uptake but no relevant changes in lactulose recovery during teduglutide treatment [110]. Concordantly, Sigalet et al. analyzed rats in an SBS model for adaptation and reported adaptation of the intestinal epithelium as measured by an increase in intestinal surface area (increased villus height) and an increase in mannitol absorption while lactulose absorption remained unaltered [111]. 

Several animal studies reported a decreased ion conductance or decreased flux of inert molecules caused by GLP-2 administration in Ussing chamber experiments in various settings. In all cases, this reduced transepithelial resistance or the decreased flux was interpreted as decreased permeability [8,112,113], but because the underlying mechanisms leading to change in transepithelial resistance in the context of GLP-2 administration are not fully understood, they harbor the risk of being misinterpreted as changes in paracellular transport. A revised interpretation is supported by Reiner et al. [84], who analyzed the impact of teduglutide administration in an SBS mouse model, where the flux of 4 kDa dextran was reduced with GLP-2 administration. The authors state that this is more likely due to the altered anatomy of hypertrophied villi rather than by changes in the paracellular pathway. It is also important to note that only mannitol recovery correlated statistically significantly with parenteral calorie requirements in SBS patients (Figure 8), which has been demonstrated in humans here. 

In further analyses, we found a correlation between mannitol recovery and the remaining small bowel length (Figure 6) in contrast to weak or absent correlation with lactulose or sucrose recovery. In humans, it is hypothesized that high urinary recovery of mannitol moieties is caused by solvent drag through pores that allow passage of mannitol but not that of lactulose. Therefore, the lactulose/mannitol ratio is primarily a standard for the normal functioning of villus epithelial cells in metabolite absorption as well as for normal villus blood flow [103]. Therefore, we conclude that in untreated SBS patients, reduced mannitol recovery is correlated with reduced enterocyte mass, while increased recovery on teduglutide treatment is correlated with an increase in enterocyte mass due to villus elongation as shown previously [25] and by increased proliferation rate (Figure 3) as well as increased blood flow [103,105,106]. Furthermore and importantly, mannitol recovery correlates significantly and inversely with parenteral calorie administration (Figure 8), which supports the concept of transcellular nutrient absorption being essentially influenced by enterocyte mass and activity, the latter being also suggested by increased SI and SGLT-1 expression (Table 3 and Figure 4). This suggests the use of 5 h urinary mannitol recovery after standardized oral ingestion as a valuable and clinically applicable functional biomarker to estimate the absorptive capacity of the gut and to monitor the improvement of small intestinal function in SBS patients. 

### 4.7. Limitations of the Studies

In SBS patients, available biomarkers assisting the interpretation of intestinal adaptation and response to GLP-2 analog treatment are extremely rare, prompting our current comprehensive comparison of potential markers. However, the presented study has some limitations. The SBS patient cohort is highly heterogeneous and includes confounders (e.g., parenteral support, unrecognized comorbidities or behavioral incompliances by the patients), which are more difficult to control in patients than in animal models. Since an attempt was made to exclude these, this led to a subset analysis with incompletely available biomaterials from some of the examined patients. Therefore, paired analysis was not always possible; however, in a substantial subset, this was possible and hitherto unpublished in humans treated with teduglutide. In this pilot study, multiple statistical comparisons were performed, which were not corrected for multiple testing, in particular in Table 3, although the biological independence of each of the tested molecules in SBS can be assumed. With adjustments for multiple testing, most of the reported results will lose statistical significance in this sub-study. Further studies with larger sample cohorts have to be performed to confirm these preliminary results. However, the important strength of the presented study is the analysis of the clinical “real world” situation in patients and, furthermore, in a subset cohort of no vs. teduglutide treatment.

## 5. Conclusions

Biomarker studies for small intestinal adaptation and treatment response to GLP-2 analogs in SBS patients are extremely limited. The impact of tight junction genes regulating paracellular transport has been discussed widely. In our study, gene expression analyses for tight junction-associated genes in SBS patients prior to and while on teduglutide treatment revealed no important or relevant impact of the paracellular route, but in contrast, altered specific gene expression was shown for genes involved in nutrient transport. Analyses of intestinal permeability in SBS patients give important insights into changes of small intestinal carbohydrate and presumably other nutrients’ absorption. Therefore, analysis of intestinal permeability proves to be applicable in SBS patients, but results have to be interpreted with the understanding that the mere use of the widely accepted calculated marker lactulose/mannitol ratio may lead to misinterpretation of results if not analyzed properly for actual measured mannitol recovery. In SBS patients, mannitol recovery does indeed correlate with remaining small bowel length and caloric parenteral requirements. Furthermore, the mannitol recovery rate was higher in patients with teduglutide treatment. Therefore, we suggest that mannitol can serve as a biomarker in order to obtain information about small intestinal absorptive processes or changes induced by GLP-2 treatment in future studies and even in clinical routine.

## Figures and Tables

**Table 1:** Patients baseline characteristics.

	Short Bowel Syndrome	Control
Number	59	19
Age (median years [range])	60 (21–88)	55 (33–86)
Sex (m/f)	21 (36%)/38 (64%)	4 (21%)/15 (79%)
Teduglutide treatment (yes/no)	41 (70%)/18 (30%)	-
Disease duration (years)	11.1 (±8.3)	
Short bowel syndrome etiology		
Vascular disease	21 (35.6%)	
Inflammatory bowel disease	13 (22.0%)	
Traumatic injury	6 (10.2%)	
Bowel obstruction	11 (18.7%)	
Cancer	3 (5.1%)	
Other (Diverticulitis, Perforation, Aganglionosis)	5 (8.5%)	
Colon continuity		
continuity	40	healthy colon
remaining small bowel length (cm)	74.2 (±41.2)	
no continuity	19	
remaining small bowel length (cm)	83.4 (±50.0)	
Parenteral support (kcal/week)	7471 (±4259)	

Data were given as absolute numbers (percentages), median with range, or as means ± SD (standard deviation).

**Table 2:** Sequence variants identified in the GLP-2 receptor gene.

Variant	Exon	Functional Domain	SNP Database	Amino Acid Exchange	Nucleotide Exchange	SIFT Score	PolyPhen Score
p.R41S	1	N-Terminus	rs114271428	Arg<Ser	AGG<AGC	0.04	0.00
p.V234I	6	Transmembrane 2	rs61730822	Val<Ile	GTC<ATC	0.22	0.003
p.D470N	13	C-Terminus	rs17681684	Asn<Asp	GAC<AAC	0.89	0.00
p.G158G	4			Gly<Gly	GAA<GAG		
p.H504H	13			His<His	CAT<CAC		
c.1-199g<a	5’UTR	pot. promoter	rs3760507	Common Polymorphism			
c.1-608a<a	5’UTR	pot. promoter	rs3760508	Common Polymorphism			
c.1-635c<g	5’UTR	pot. promoter	no entry				
c.1-770c<g	5’UTR	pot. promoter	rs2047664	Common Polymorphism			

**Table 3:** Relative gene expression in untreated SBS patients compared to healthy controls and SBS couples prior to treatment and while on teduglutide treatment, including patient characteristics at the bottom of the table.

	Relative Gene Expression			Relative Gene Expression (*n* = 18)		
Gene	Control (*n* = 7)	SBS-(Untreated) (*n* = 37)	*p*-Value ^§^	SBS Prior Treatment	SBS on TED	*p*-Value ^#^
	Mean (±SD)	Mean (±SD)		Mean (±SD)	Mean (±SD)	
Tight junction						
*CLDN10*	0.0007 (±0.001)	0.0008 (±0.0017)	0.66	0.0003 (±0.0017)	0.0001 (±0.0002)	0.27
*CLDN15*	0.0103 (±0.0118)	0.0132 (±0.014)	0.75	0.0132 (±0.017)	0.016 (±0.01)	0.30
*OCLN*	0.0028 (±0.0009)	0.0026 (±0.0011)	0.79	0.0025 (±0.0009)	0.0029 (±0.0015)	0.55
*ZO-1*	0.0514 (±0.045)	0.0357 (±0.031)	0.21	0.031 (±0.015)	0.033 (±0.008)	0.73
*CRB3*	0.013 (±0.003)	0.015 (±0.008)	0.80	0.0158 (±0.016)	0.0128 (±0.0028)	0.68
*CASK*	0.053 (±0.012)	0.043 (±0.014)	**0.006**	0.0389 (±0.013)	0.054 (±0.014)	**0.009**
Nutrient transport						
*SI*	0.72 (±0.023)	0.477 (±0.313)	**0.030**	0.417 (±0.296)	0.673 (±0.311)	**0.024**
*SGLT1*	0.262 (±0.082)	0.211 (±0.145)	0.30	0.169 (±0.127)	0.309 (±0.146)	**0.002**
Proliferation						
*MKI67*	0.0066 (±0.002)	0.00723 (±0.005)	0.93	0.0071 (±0.0039)	0.0069 (±0.0052)	0.78
Structure						
*CK20*	0.534 (±0.201)	1.05 (±1.55)	0.93	1.0 (±1.2)	0.449 (±0.181)	0.06
Citrulline (µmol/L)	-	26.8 (±17.7)		23.0 (±14)	42.2 (±22.3)	**<0.001**
kcal/week	-	7755 (±3818)		7255 (±3928)	3362 (±4342)	**<0.001**
rSBL (cm)	-	77.2 (±42.1)		68.8 (±38.3)	68.8 (±38.3)	-

Relative gene expression was calculated with the ΔCt method and given as 2^−ΔCt^ (∆Ct = Ct*GOI* − Ct*HKG*). *CLDN10*, Claudin 10; *CLDN15*, Claudin 15; *OCLN*, Occludin; *ZO-1*, zonula occludens protein 1; *CRB3*, crumbs 3, cell polarity complex component; *CASK*, Calcium/calmodulin-dependent serine protein kinase; *SI*, sucrase-isomaltase; *SGLT-1*, Sodium-dependent glucose transporter 1; *MKI67*, marker of proliferation associated antigen Ki-67; *CK20*, cytokeratin 20. Bold *p*-values indicate values below *p* < 0.05. Statistical calculation was not corrected for multiple testing. Considering the high number of simultaneous statistical comparisons, significance will get lost with the appropriate correction for multiple testing. ^§^ Mann–Whitney, ^#^ Wilcoxon.

## Data Availability

The data presented in this study are available on request from the corresponding author. The data are not publicly available due to institutional guidelines.

## Abbreviations

SBS: Short bowel syndrome, GLP-2: Glucagon-like peptide-2, GLP-2R: Glucagon-like-peptide-2 receptor, TED: teduglutide, rSBL: remaining small bowel length, IP: Intestinal permeability.
